# The Secretion of Inflammatory Cytokines Triggered by TLR2 Through Calcium-Dependent and Calcium-Independent Pathways in Keratinocytes

**DOI:** 10.1155/mi/8892514

**Published:** 2024-11-16

**Authors:** Eun-Ok Kim, Dain Park, In Jin Ha, Se-Eun Bae, Min Young Lee, Miyong Yun, Kyuseok Kim

**Affiliations:** ^1^Medical Science Research Center, College of Medicine, Korea University, Seoul 02841, Republic of Korea; ^2^Korean Medicine Clinical Trial Center, Kyung Hee University Korean Medicine Hospital, Seoul 02447, Republic of Korea; ^3^Department of Anatomy, College of Medicine, Korea University, Seoul 02841, Republic of Korea; ^4^Department of Bioindustry and Bioresource Engineering, Sejong University, Seoul 05006, Republic of Korea; ^5^Department of Ophthalmology, Otolaryngology and Dermatology of Korean Medicine, College of Korean Medicine, Kyung Hee University, Seoul 02447, Republic of Korea

## Abstract

Keratinocytes can be activated by *Cutibacterium acnes*, leading to the production of proinflammatory cytokines via toll-like receptors (TLRs) 2 and 4. Although several studies have investigated keratinocytes, the mechanism of calcium-mediated activation remains unclear. Herein, we investigated whether calcium influx via TLR2 and TLR4 stimulation was involved in cytokine secretion by keratinocytes in HaCaT cells. Although TLR2 stimulation by peptidoglycan (PGN) increased intracellular calcium influx, TLR4 stimulation by lipopolysaccharide (LPS) did not increase it, as analyzed using flow cytometry with the calcium indicator Fluo-3. However, activation by either TLR2 or TLR4 ligands upregulated the intracellular calcium influx in THP-1 monocytes. Additionally, the expression of major proinflammatory cytokines and chemokines, such as interleukin (IL)-6, IL-8, IL-1*α*, granulocyte-macrophage colony-stimulating factor (GM-CSF), and monocyte chemoattractant protein-1 (MCP-1), was significantly increased by TLR2 in HaCaT cells. Moreover, treatment with the intracellular calcium chelator, BAPTA-AM, disrupted PGN-mediated induction of IL-6, IL-8, and MCP-1 production. Real-time quantitative polymerase chain reaction (PCR) and western blotting revealed that TLR2 stimulation induced expression of the epidermal differentiation marker keratin 1. In conclusion, TLR2-induced intracellular calcium influx plays a pivotal role in the secretion of proinflammatory cytokines, such as IL-6 and MCP-1, in keratinocytes. Moreover, the continuous influx of calcium via TLR2 activation leads to keratinization. In vitro studies using HaCaT cells provide basic research on the effect of TLR2-induced calcium on *C. acnes*-mediated inflammation in keratinocytes. These studies are limited in their ability to clinically predict what happens in human keratinocytes. Clinical studies on patients with acne, including three-dimensional (3D) cultures of primary keratinocytes, are required to develop new diagnostic markers for determining the severity of acne vulgaris.

## 1. Introduction

Acne vulgaris is a common skin disorder of the pilosebaceous follicle characterized by noninflammatory or inflammatory lesions, such as open or closed comedones, papules, pustules, nodules, or cysts. The exact cause and mechanism of acne remain unknown but may be due to multifactorial factors, including androgen-induced excess sebum production, ductal epidermal hyperkeratinization, and colonization by the bacterium *Cutibacterium acnes* (formerly *Propionibacterium*) [1]. *C. acnes* specifically causes inflammatory acne. *C. acnes* induces the production of inflammatory cytokines, including tumor necrosis factor-*α*, interleukin (IL)-1*β*, IL-8, and IL-6, by keratinocytes, sebocytes, monocytes, and macrophages via toll-like receptors (TLRs) 2 and 4 [2–11]. Keratinocytes are the main cell population in the human epidermis involved in the initial physical and immunological protection of the epidermis [12]. The constitutive expression of TLR2 and TLR4 was demonstrated in human epidermal keratinocytes and is stimulated by exposure to specific TLR ligands [13–16], leading to the activation of specific signaling cascades, including the nuclear factor κB, activator protein 1, and mitogen-activated protein kinase [6, 11, 17–20], resulting in increased expression of proinflammatory cytokines [21]. Particularly, peptidoglycan (PGN), a major component of the cell wall of *C. acnes*, a gram-positive bacterium, produces inflammation-related cytokines via TLR2 activation [6, 11, 18, 22], whereas lipopolysaccharide (LPS) from gram-negative bacteria is produced via TLR4 activation [23–25]. Therefore, modulation of TLR2 expression has been proposed as a novel target for acne treatment [18, 26, 27].

Routine monitoring of the inflammatory responses by culturing primary human keratinocytes in vitro has major drawbacks, including the requirement of supplementary growth factors for cell survival and proliferation, a short lifespan, interindividual diversity of growth characteristics and in vitro responses, and changes in the proliferation and differentiation properties with increasing passage numbers. To minimize these issues, HaCaT cells have been proposed as a model for functional keratinocyte studies. HaCaT cells are nontumorigenic monoclonal cells that can survive for long periods in the absence of supplemental growth factors. They also exhibit normal cellular morphology and express all major surface markers and functional activities [28]. Therefore, HaCaT cells are the most widely used cell line for studying skin barrier homeostasis and differentiation [28, 29]. Monocytes, one of the innate immune cells, are also activated by *C. acnes* and are known to be able to secrete several inflammatory cytokines, such as TNF-*α*, IL-6, IL-8, and IL-1*β* [2]. Calcium is a known chemokinetic agent in circulating human monocytes [30], and THP-1 cells have been used in vitro to analyze the activity of monocytes induced by acne [31].

Ionic distribution plays a crucial role in the maintenance of skin homeostasis. Calcium ions (Ca^2+^) are important molecules distributed in the epidermis and involved in the activation of immune cells, skin barrier homeostasis [32–34], and keratinocyte differentiation [35]. Keratinization of the epidermal barrier caused by *C. acnes* induces the infiltration of bacteria into the epidermis, leading to the occurrence and worsening of acne. Cell culture supernatants of *C. acnes* induced cytokine secretion through intracellular calcium signaling in keratinocytes [36]. However, the association between calcium influx induced by *C. acnes* and cytokine secretion by keratinocytes remains unclear. In this study, we focused on the correlation between TLR2 activation and calcium influx induced by *C. acnes*. Additionally, the effect of calcium influx on the production of inflammatory cytokines via TLR2 and TLR4 in immortalized human keratinocytes (HaCaT cells) and human monocytic cell line (THP-1), respectively.

## 2. Materials and Methods

### 2.1. Cell Cultures and Reagents

HaCaT cells were cultured in calcium-free Dulbecco's Modified Eagle Medium (DMEM; HyClone, Piscataway, NJ, USA) supplemented with 4 mM L-glutamine (Gibco, Thermo Fisher Scientific, MA, USA), 1 mM sodium pyruvate (Gibco, Thermo Fisher Scientific), 10% heat-inactivated fetal bovine serum (FBS; Hyclone), and 1% antibiotic/antimycotic solution (Gibco, Thermo Fisher Scientific). Stably differentiated HaCaT cells were maintained in high-calcium DMEM (HyClone) for analysis. The human monocyte cell line THP-1 was cultured in RPMI 1640 (PAN-Biotech, Aidenbach, Germany) supplemented with 25 mM 4-(2-hydroxyethyl)-1-piperazineethanesulfonic acid (HEPES) (Gibco, Thermo Fisher Scientific), 10% heat-inactivated FBS, and 1% antibiotic/antimycotic solution. All cells were cultured in a humidified incubator with 5% CO_2_ at 37°C. LPS (Sigma–Aldrich, St. Louis, MO, USA) from *Escherichia coli* 055: B5 and PGN (Sigma–Aldrich) from *Staphylococcus aureus* were dissolved in distilled water and used as TLR4 and TLR2 stimulators, respectively [37, 38]. BAPTA-AM, an intracellular calcium chelator, was purchased from Abcam (Cambridge, MA, USA). The TLR4- and TLR2-specific inhibitors, CLI-095 and TL2-C29, respectively, were purchased from InvivoGen (San Diego, CA, USA). To measure keratin 1 levels, HaCaT cells were cultured in DMEM (PAN-Biotech). Cells were treated with PGN (50 µM) for the indicated times and analyzed.

### 2.2. Multiplex Analysis of Cytokines

Cells were incubated with PGN (5 µM) or LPS (100 nM) for 24 h in six-well plates. BAPTA-AM was used with PGN in certain experiments. The concentrations of human cytokines and chemokines (IL-6, IL-1*α*, granulocyte-macrophage colony-stimulating factor [GM-CSF], C-X-C motif chemokine ligand 8/IL-8, and C─C motif chemokine ligand 2/monocyte chemoattractant protein-1 [MCP-1]) in cell culture supernatants were measured using the Human Premixed Multi-Analyte Kit (R&D Systems, Minneapolis, MN, USA) according to the manufacturer's instructions. Data were collected using the Luminex 200 software (Luminex, Austin, TX, USA), and the concentration of each cytokine was automatically calculated using the MILLIPLEX Analyst 5.1 software. Standard curves for each cytokine were generated using reference cytokines provided by the kit. Data are represented as the mean ± standard deviation (SD) of at least two independent experiments performed in triplicate.

### 2.3. Real-Time Quantitative Polymerase Chain Reaction (PCR)

Total RNA was isolated using Purehelix RNA Extraction Solution (NANOHELIX, Daejeon, Republic of Korea), according to the manufacturer's instructions. complementary DNA (cDNA) was synthesized using the PrimeScript 1st strand cDNA Synthesis Kit (Takara Korea Biomedical Inc., Seoul, Republic of Korea), according to the manufacturer's instructions. The amplification of each cDNA sample/library was monitored using the Sensi FAST SYBR Hi-ROX kit (Bioline, Taunton, MA, USA) on a StepOnePlus (Applied Biosystems, Foster, CA, USA). The following specific primers were used: keratin 1, forward: 5′-CCA GGA GCT GAT GAA CAC CAA-3′ and reverse: 5′-GAG GGT CCT GTA GGT GGC AAT-3′; IL-6, forward: 5′-AGT GAG GAA CAA GCC AGA GC-3′ and reverse: 5′-GGT CAG GGG TGG TTA TTG CA-3′; IL-1*α*, forward: 5′-TCT TCT GGG AAA CTC ACG GC-3′ and reverse: 5′-CCA GAC CTA CGC CTG GTT TT-3′; GM-CSF, forward: 5′-AAC CCC GGA AAC TTC CTG TG-3′ and reverse: 5′-GTA TCA GGG TCA GTG TGG CC-3′; IL-8, forward: 5′-TCA GAG ACA GCA GAG CAC ACA A-3′ and reverse: 5′-GTT CCT TCC GGT GGT TTC TTC-3′; and MCP-1, forward: 5′-CAG ATG CAA TCA ATG CCC CAG T-3′ and reverse: 5′-ATA AAA CAG GGT GTC TGG GGA AAG C-3′. Additionally, glyceraldehyde-3-phosphate dehydrogenase (GAPDH) was used as an internal control. Data are represented as the mean ± SD of at least three independent experiments performed in triplicate.

### 2.4. Western Blotting

Cells were harvested, and the total protein was prepared by lysing the cells with lysis buffer (1M Tris-HCl [pH 7.4], 5M NaCl, 0.5M ethylenediaminetetraacetic acid [EDTA], 10% glycerol, 1% Triton X-100 supplemented with 1 mM phenylmethylsulfonyl fluoride [PMSF], 1 mm Na_3_Vo_4_, and 50 mm NaF). Protein extract was diluted using 5× sample buffer and denatured at 95°C for 5 min. Samples were electrophoresed on 10% sodium dodecyl sulfate-polyacrylamide gel electrophoresis (SDS-PAGE) gels and transferred onto 0.45-μm nitrocellulose membranes (GE Healthcare Life Sciences, Chicago, IL, USA). Membranes were blocked for 1 h at room temperature with 5% skim milk in tris-buffered saline with Triton X-100 containing 0.05% Tween 20 (tris-buffered saline with Tween 20 [TBST]) and incubated overnight at 4°C with the following primary antibodies: keratin-1 (1:1000, BioLegend, San Diego, CA, USA) and *β*-actin (1:2000, Santa Cruz Biotechnology, Dallas, TX, USA). Subsequently, the membranes were incubated with the appropriate horseradish peroxidase-conjugated secondary antibodies at room temperature for 1 h and washed three times with TBST. The membranes were then subjected to chemiluminescence-based detection. A ChemiDoc system and iBright Analysis software (CL1000; Invitrogen, Thermo Fisher Scientific) were used to analyze band intensity. Quantitative differences were determined by densitometry using the ImageJ 1.8.0 software (https://imagej.nih.gov/ij/) and normalized to the *β*-actin signal. Data are presented as mean ± SD from at least three independent experiments.

### 2.5. Measurement of Ca^2+^ Mobilization

Calcium mobilization was measured using Fluo-3 AM, a membrane-permeable Ca^2+^-sensitive fluorescent dye. Cells (5 × 10^6^) were resuspended in Hankʼs balanced salt solution (HBSS) buffer and loaded with 5 µM Fluo-3 AM (Molecular Probes, Eugene, OR, USA) in the presence of Pluronic F-127 (Biotium, Fremont, CA, USA) and incubated at 37°C for 30 min. The cells were then washed twice with HEPES buffer and stored on ice. Cells were analyzed using a FACSCanto II flow cytometer (BD Biosciences, San Jose, CA, USA). Data were collected using the FACSDiva software (BD Biosciences) to detect Ca^2+^ influx. Basal calcium influx was analyzed using a flow cytometer for 3 min, followed by adding 5 µM PGN or 100 ng/mL LPS, and data were collected for another 5 min. Ionomycin (1 µg/mL; Sigma–Aldrich) was used as a positive control. The mean fluorescence over time was analyzed using FlowJo software (LLC, OR, USA). At least three independent experiments were conducted.

### 2.6. Flow Cytometry Analysis

Cells were suspended in 100 µL of FACS buffer (phosphate-buffered saline with 2 mM EDTA and 5% FBS) and incubated with anti-TLR2-Alexa 488 (BD Biosciences) and anti-TLR4-PE (BD Biosciences) antibodies according to the manufacturer's recommendations. Cells were analyzed using a FACSCanto II flow cytometer, and data were collected using the FACSDiva software. Data were analyzed using the FlowJo software to compare expression levels.

### 2.7. Statistical Analysis

Statistical analyses were performed using SigmaPlot 10.0 software (Systat Software, Inc., San Jose, CA, USA). An unpaired Student's *t*-test was used to compare mock and LPS or PGN treatments to calculate the *p*-value [39]. Results were considered statistically significant at *p* < 0.05.

## 3. Results

### 3.1. Intracellular Calcium Uptake by PGN-Induced TLR2 Stimulation in Keratinocytes

**Figure 1:**
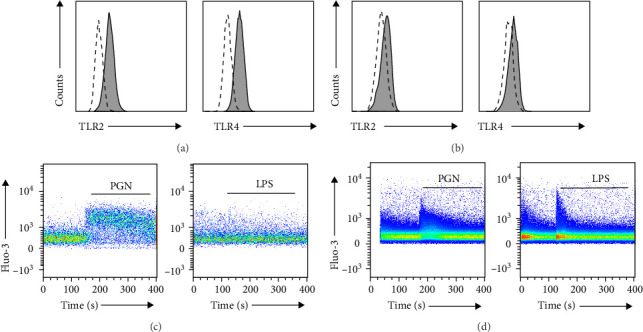
Increased intracellular calcium influx following PGN treatment in HaCaT cells. Cell surface expression of TLR2 and TLR4 on HaCaT (a) and THP-1 cells (b) was measured using flow cytometry. Filled histograms refer to TLR2 and TLR4, and dotted histograms refer to isotype control IgG. Intracellular calcium influx was measured in HaCaT cells (c) and THP-1 cells (d) labeled with the calcium-specific indicator Fluo-3. Basal calcium levels were measured, and then calcium influx was induced through treatment with 5 µg/mL of PGN or 100 ng/mL of LPS (red line). Intracellular calcium influx was measured over time using a FACS Canto II flow cytometer. LPS, lipopolysaccharide; PGN, peptidoglycan.

The cell surface expression of TLR2 and TLR4 was confirmed in immortalized keratinocytes, HaCaT cells, THP-1 cells, and a human monocytic cell line, using flow cytometry (Figures 1a,b). Based on these results, the correlation between TLR2 and TLR4 and intracellular calcium influx was analyzed in immortalized keratinocytes, HaCaT cells, and THP-1 monocytic cells. To activate TLR2 and TLR4, PGN and LPS, which are specific agonists of the receptors, respectively, were used, and intracellular calcium mobilization was measured using flow cytometry with Fluo-3, a calcium indicator. The activation of TLR2 and TLR4 in HaCaT and THP-1 cells showed different results. In HaCaT cells, calcium influx increased specifically upon PGN treatment, whereas no such increase was observed upon LPS treatment (Figure 1c). When the LPS concentration was increased to 1000 ng/mL, no increase in intracellular calcium was observed (Figure S1). In contrast, THP-1 cells showed increased intracellular calcium levels after treatment with either PGN or LPS (Figure 1d). These results suggested that the mechanism of calcium influx via TLR2 or TLR4 activation differed between keratinocytes and monocytes. In particular, TLR2 activation by PGN, a major cell wall component of *C. acnes*, is important for increasing intracellular calcium levels in keratinocytes.

### 3.2. Abnormal Keratinization by PGN-Induced TLR2 Stimulation in Keratinocytes

**Figure 2:**
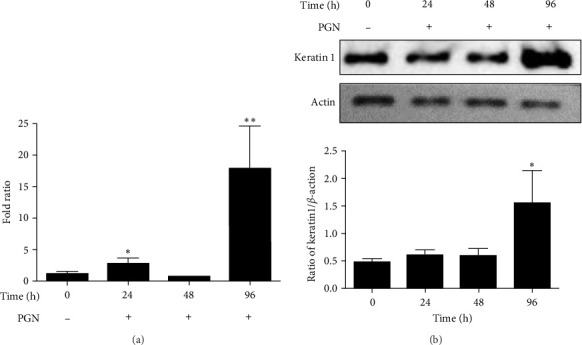
Increased expression of keratin 1 following PGN treatment in HaCaT cells. (a) HaCaT cells were treated with 50 µg/mL of PGN for 96 h, and the expression of keratin 1 was confirmed at the RNA level. (b) Keratin 1 protein levels by western blot. All experiments were performed in triplicate. Data are representative of three experiments. Data are mean ± SD. ⁣^*∗*^*p* < 0.05, ⁣^*∗∗*^*p* < 0.01 versus control HaCaT cells. PGN, peptidoglycan.

Keratinocytes undergo differentiation when exposed to a high-calcium environment for prolonged periods [40, 41]. Increased intracellular Ca^2+^ concentrations lead to abnormal keratinization in cultured human keratinocytes [42]. The effect of PGN-induced TLR2 activation on the keratinization of HaCaT cells was investigated by analyzing the expression of the keratinization differentiation marker, keratin 1. A slight increase in the RNA level of keratin 1 was observed at 24 h after PGN treatment, followed by a significant increase of ~15-fold at 96 h (Figure 2a). The changes in keratin 1 protein also showed a similar pattern to the RNA expression results, with a significant increase of approximately threefold at 96 h after PGN treatment (Figure 2b).

### 3.3. Involvement of Calcium-Dependent IL-6 and MCP-1 Secretion in PGN-Induced TLR2 Stimulation in Keratinocytes

**Figure 3:**
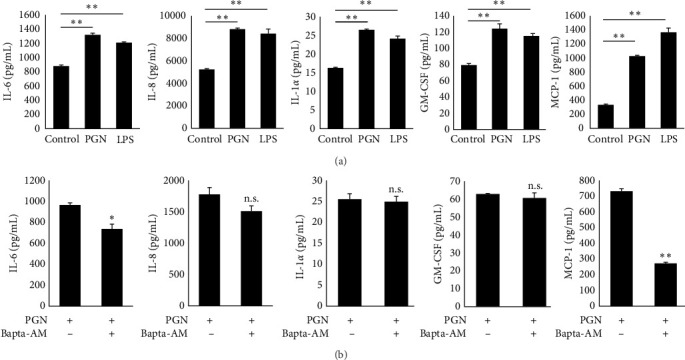
Expression levels of IL-6, IL-8, IL-1*α*, GM-CSF, and MCP-1 after treatment with PGN or BAPTA-AM in HaCaT cells. (a) HaCaT cells were treated with 5 µg/mL of PGN or 100 ng/mL of LPS and cultured for 24 h. Culture supernatants were harvested, and cytokine and chemokine secretion was measured using a multiplex. The untreated group was used as a control. (b) HaCaT cells were treated with PGN or BAPTA-AM and PGN for 24 h, culture supernatants were harvested, and secreted cytokines and chemokines were analyzed using multiplex. All experiments were performed in triplicate. Data are mean ± SD. n.s.: non-significant, ⁣^*∗*^*p* < 0.05, ⁣^*∗∗*^*Pp* < 0.01 versus control. GM-CSF, granulocyte-macrophage colony-stimulating factor; LPS, lipopolysaccharide; PGN, peptidoglycan.

The secretion of inflammatory cytokines and chemokines was analyzed based on differences in calcium influx between TLR2 and TLR4 in HaCaT cells. The secretion of cytokines, such as IL-1*α*, IL-6, and GM-CSF, and chemokines, such as MCP-1 and IL-8, known to be secreted by keratinocytes [43], were significantly increased after PGN and LPS treatment (Figure 3a). However, there was no change in the levels of tumor necrosis factor-*α* (data not shown). To confirm that the increase in cytokines was specifically linked to TLR2 and TLR4, selective inhibitors were used: TL-2 C29, a potential TLR2 inhibitor [44], and CLI-095, a potential TLR4 inhibitor [45]. The secretion of IL-6, IL-8, IL-1*α*, GM-CSF, and MCP-1, which were elevated by PGN and LPS treatment, was reduced by both TL-2 C29 and CLI-095 (Figure S2). To investigate the effect of PGN-induced TLR2 stimulation on calcium signaling mechanisms and cytokine and chemokine production, calcium influx was inhibited using the calcium chelator BAPTA-AM. Inhibition of calcium uptake by BAPTA-AM drastically reduced IL-6 and MCP-1 production among the cytokines and chemokines that were increased by PGN (Figure 3b). In addition, IL-8 production was reduced; however, the difference was not statistically significant. Moreover, there were no significant changes in IL-1*α* and GM-CSF levels. These results demonstrate that IL-6 and MCP-1 production via PGN-induced TLR2 stimulation was achieved in an intracellular calcium-dependent manner in keratinocytes, and IL-1*α*, GM-CSF, and IL-8 were produced through a calcium-independent pathway.

## 4. Discussion

Bacterial pathogens release Ca^2+^ from their intracellular stores by activating TLR2 and TLR4, which are required for the activation of proinflammatory cytokine gene expression [46, 47]. This suggests calcium is a secondary messenger that modulates the proinflammatory response during bacterial infection. A recent report showed that the culture supernatant of *C. acnes* can induce cytokine secretion via intracellular calcium signaling in keratinocytes [36]. In our study, in contrast to THP-1 cells, we observed that calcium influx was mediated by TLR2 and not by TLR4 in HaCaT-immortalized keratinocytes. Macrophages and keratinocytes use different mechanisms for calcium influx [46, 47]. Based on these results, we hypothesized that the production of cytokines and chemokines induced by *C. acnes* in keratinocytes is involved in TLR2-mediated intracellular calcium influx. We observed a significant increase in the production of cytokines and chemokines, such as IL-6, IL-8, and MCP-1, following PGN-induced stimulation of TLR2. Among these cytokines released upon TLR2 stimulation, IL-6 and MCP-1 secretion is regulated in a calcium-dependent manner, whereas IL-8, IL-1*α*, and GM-CSF are produced in a calcium-independent manner. TLR1–6 and TLR9 are expressed on keratinocytes, and most TLRs on keratinocytes are expressed as homodimers. TLR2 exists as a heterodimer with TLR1 or TLR6 [48]. TLR1/TLR2 recognize triacylated lipopeptides, and TLR2/TLR6 recognize diacylated lipopeptides. In contrast to other gram-positive bacteria, the PGN of *C. acnes* is the only ligand of TLR2. Calcium influx plays an important role in the signal transduction mechanism of TLR2. TLR2-triggered calcium entry induces the phosphorylation of several signaling adaptor proteins, such as phosphoinositide 3-kinase and phospholipase *Cɤ* [49], which are involved in the producing inflammatory cytokines, such as IL-6 or MCP-1. Furthermore, the intracellular concentration of calcium ions affects the keratinization of mouse and human keratinocytes by disrupting desmosome formation [35, 50].

Upon activation, TLR2 can influence the function of the endoplasmic reticulum (ER) and mitochondria, leading to function change, such as calcium release from the ER [51] or the production of reactive oxygen species from mitochondria [52]. This can further exacerbate mitochondrial dysfunction and contribute to ER stress. Mitochondria and ER regulate intracellular calcium levels [53, 54]. When either organelle is damaged, calcium homeostasis can be disrupted, leading to excessive cytosolic calcium levels. The interplay between TLR2, ER, mitochondria, and calcium signaling is crucial for regulating immune response and maintaining cellular homeostasis. Dysregulation in these pathways can lead to inflammatory cytokines.

Inflammatory cytokines and chemokines in keratinocytes are involved in the recruitment and activation of inflammation-related immune cells, such as macrophages and neutrophils, and this cascade of responses exacerbates inflammation. MCP-1 is a major chemoattractant for macrophages and monocytes [55]. An increase in the number of macrophages in the skin has been reported in the early stages of acne lesions [56], which can be explained by the elevated secretion of MCP-1 by keratinocytes. This suggests that macrophages that accumulate in the skin due to MCP-1 play a prominent role in the development of acne lesions. In addition, IL-6 plays a crucial role in various inflammatory skin diseases [57]. IL-6 is a key regulator of the inflammatory response and primarily produced in epidermal keratinocytes following damage caused by chronic skin diseases or bacterial infections. IL-6 induces the release of several proinflammatory cytokines from tissue-resident macrophages and keratinocytes, and it chemoattracts leukocytes to the tissues, leading to tissue repair through the immune response [58]. IL-6 can also cause abnormal differentiation of dermal tissue [59].

HaCaT cells have been widely used to study the pathology of skin keratinocytes in infectious diseases and tumors. In vitro studies with HaCaT cells have shown advantages, such as reproducibility, reliability, and lower interindividual variability compared with those of keratinocytes isolated from skin biopsies. However, one limitation is the inability to reproduce the complexity and cellular heterogeneity of the skin under basal or inflammatory conditions. To address these issues, a three-dimensional (3D) culture system using HaCaT cells has recently been developed [60, 61]; however, further testing is needed to fully depict the skin structure and environment. In this study, we observed that keratinocytes secreted IL-6 and MCP-1 during acne induction through a TLR2-calcium-related signaling mechanism distinct from that of macrophages. Therefore, the regulation of intracellular calcium concentration by TLR2 should be considered in treating inflammatory acne.

## 5. Conclusions

The relationship between the infectious process of *C. acne*s and calcium ions may play an important role in diagnosing acne vulgaris. Therefore, studies are underway to measure the calcium concentration in the serum of patients with acne. Based on this study, the calcium ion concentration corresponding to TLR2 expression level could be used as a new diagnostic index for examining the severity of acne.

## Data Availability

The data that support the findings of this study are available on request from the corresponding author.

## Supporting Information

Supporting Information Figure S1. Intracellular calcium influx following LPS treatment comprehensive image analysis. Figure S2. Inhibition of cytokine production following TL-2 C29 and CLI-95.
