# Supplementary figures and images for: The Secretion of Inflammatory Cytokines Triggered by TLR2 Through Calcium-Dependent and Calcium-Independent Pathways in Keratinocytes
Source: Mediators Inflamm. 2024 Nov 16;2024:8892514. doi: 10.1155/mi/8892514 (PMC11588404; doi:10.1155/mi/8892514)

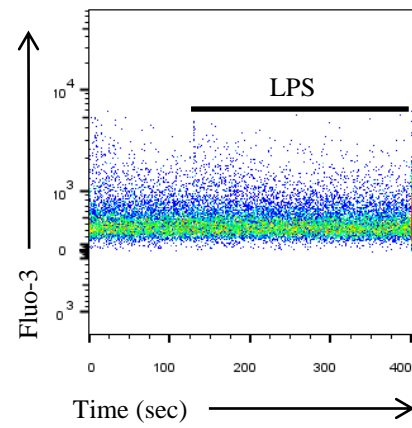

Figure S1

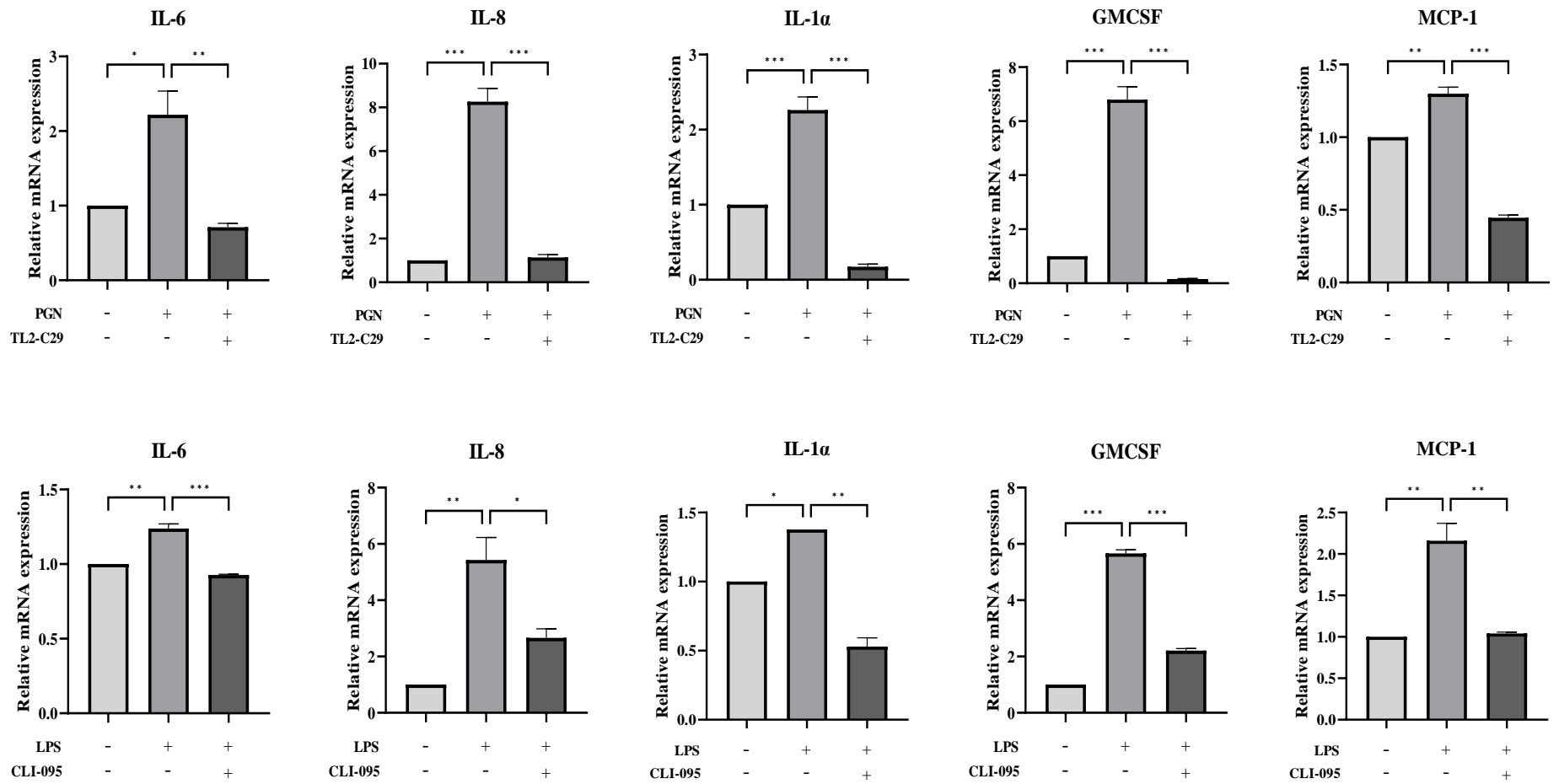

Figure S2

Supplement: Supporting Information — Figure S1. Intracellular calcium influx following LPS treatment comprehensive image analysis. Figure S2. Inhibition of cytokine production following TL-2 C29 and CLI-95. [file 8892514.f1.pdf]
